# VAN: an R package for identifying biologically perturbed networks via differential variability analysis

**DOI:** 10.1186/1756-0500-6-430

**Published:** 2013-10-25

**Authors:** Vivek Jayaswal, Sarah-Jane Schramm, Graham J Mann, Marc R Wilkins, Yee Hwa Yang

**Affiliations:** 1 School of Mathematics and Statistics, The University of Sydney, Sydney, NSW, Australia; 2 Westmead Millennium Institute for Medical Research, Sydney Medical School, The University of Sydney, Sydney, NSW, Australia; 3 Melanoma Institute Australia, Sydney, NSW, Australia; 4 School of Biotechnology and Biomolecular Sciences, University of New South Wales, Sydney, NSW, Australia; 5 Systems Biology Initiative, University of New South Wales, Sydney, NSW, Australia

**Keywords:** Protein-protein interaction networks, Network modules, Melanoma

## Abstract

**Background:**

Large-scale molecular interaction networks are dynamic in nature and are of special interest in the analysis of complex diseases, which are characterized by network-level perturbations rather than changes in individual genes/proteins. The methods developed for the identification of differentially expressed genes or gene sets are not suitable for network-level analyses. Consequently, bioinformatics approaches that enable a joint analysis of high-throughput transcriptomics datasets and large-scale molecular interaction networks for identifying perturbed networks are gaining popularity. Typically, these approaches require the sequential application of multiple bioinformatics techniques – ID mapping, network analysis, and network visualization. Here, we present the Variability Analysis in Networks (VAN) software package: a collection of R functions to streamline this bioinformatics analysis.

**Findings:**

VAN determines whether there are network-level perturbations across biological states of interest. It first identifies hubs (densely connected proteins/microRNAs) in a network and then uses them to extract network modules (comprising of a hub and all its interaction partners). The function *identifySignificantHubs* identifies dysregulated modules (i.e. modules with changes in expression correlation between a hub and its interaction partners) using a single expression and network dataset. The function *summarizeHubData* identifies dysregulated modules based on a meta-analysis of multiple expression and/or network datasets. VAN also converts protein identifiers present in a MITAB-formatted interaction network to gene identifiers (UniProt identifier to Entrez identifier or gene symbol using the function *generatePpiMap*) and generates microRNA-gene interaction networks using TargetScan and Microcosm databases (*generateMicroRnaMap*). The function *obtainCancerInfo* is used to identify hubs (corresponding to significantly perturbed modules) that are already causally associated with cancer(s) in the Cancer Gene Census database. Additionally, VAN supports the visualization of changes to network modules in R and Cytoscape (*visualizeNetwork* and *obtainPairSubset*, respectively). We demonstrate the utility of VAN using a gene expression data from metastatic melanoma and a protein-protein interaction network from the Human Protein Reference Database.

**Conclusions:**

Our package provides a comprehensive and user-friendly platform for the integrative analysis of -omics data to identify disease-associated network modules. This bioinformatics approach, which is essentially focused on the question of explaining phenotype with a 'network type’ and in particular, how regulation is changing among different states of interest, is relevant to many questions including those related to network perturbations across developmental timelines.

## Findings

### Introduction

Network based approaches for analyzing -omics data are necessitated by the daunting intricacies of biomolecular systems and have the potential to quantifiably model large-scale functional networks [1]. These approaches can be broadly divided into two categories – generating novel networks by analyzing -omics data [2-4] and using pre-defined networks to analyze -omics data *e.g.*, [5-7]. Our paper focuses on the latter approach and describes a method for the identification of network modules *i.e.* a hub and all its interaction partners that are perturbed across biological conditions. Hubs are of special relevance to medicine and disease because they are both the source of network robustness to failure, as well as its weakness (discussed in detail in [8]). Moreover, proteins with 6 to 38 interaction partners are frequently observed among existing cancer therapeutic targets [9]. To better understand how protein networks may act to control biological responses, the ongoing development of tools to analyze relationships between network structure and function is important.

A network-based analysis of transcriptomics data differs from the more conventional analysis of transcriptomics data. The latter relies on methods that were developed for the identification of differentially expressed genes or gene sets (*e.g.,*[10]). These methods evaluate changes in the expression levels of genes across biological states rather than changes in the strength of gene-gene correlations across biological states. Given that a disease is often a consequence of localized or large-scale perturbations in the strength of molecular interactions rather than changes in individual genes [11] there is a need for methods that focus on gene-gene correlations. Such methods examine whether, for a given network module, the average gene expression correlation between a hub (*i.e.* a densely-connected node) and its interaction partners is the same among two biological conditions. This approach was initially proposed by Han and colleagues in yeast [12] to measure the correlation (tightness) within a network module and was subsequently applied by Taylor *et al.*[5] to analyse breast cancers in a study that also included examination of the prognostic utility of such modules. The current approach by Taylor *et al.*[5] has two main limitations. First, it does not account for variability in the results owing to differences in the expression and/or network datasets used. Second, the test statistic is limited to the analysis of two biological conditions of interest. In addition to these issues, there is a growing requirement for the availability of user-friendly software, suitable for a broad community of end-users, for the implementation of network-centric analyses.

In this paper, we introduce a data analysis pipeline for the identification of dysregulated network modules using one or more transcriptomic datasets and molecular interaction networks. If multiple datasets or networks are provided as input to our pipeline, then we provide an end-user the option of integrating the results using meta-analysis approaches. We illustrate the benefit of our pipeline using a publicly available melanoma dataset and protein-protein interaction (PPI) dataset and identify hubs of potential relevance to melanoma biology.

## Methods

**Figure 1:**
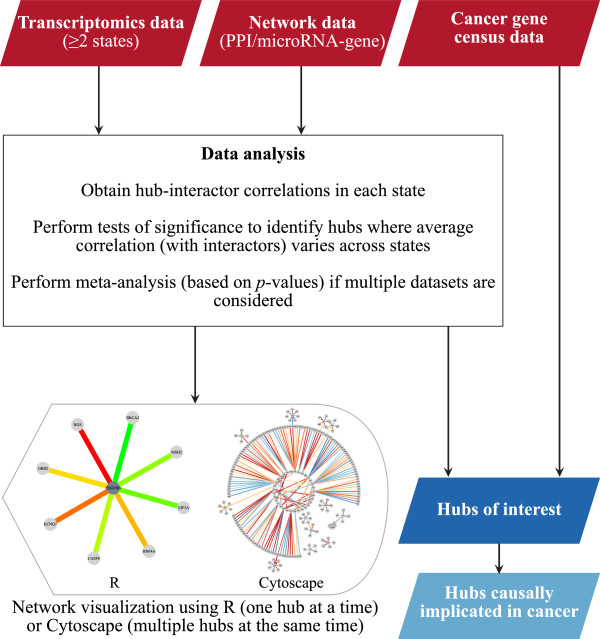
VAN pipeline for the identification of hubs that are significantly perturbed across biological states.

Variability Analysis in Networks (VAN) provides a suite of tools for testing and visualizing the dysregulation of modules in molecular interaction networks (Figure 1 see also Additional file 1 – VAN User Guide).

### Significant network modules

VAN enables an integrative analysis of (a) gene expression data with PPI network data or (b) gene and microRNA expression data with microRNA-gene interaction network data using the function *identifySignificantHubs*. Firstly, VAN identifies the hubs where the number of interaction partners, which are present in both the network and expression data, is greater than a user-defined threshold value *e.g.*, a hub may be defined as a gene/microRNA with at least five interaction partners. Each hub, along with its interaction partners, represents a network module. Secondly, VAN calculates the correlation of gene expression between the hub and each of its interaction partners in every biological state of interest. Thirdly, it generates the statistic for testing the null hypothesis that the average correlation is the same in all the biological states. The test statistic is similar to that defined by [5] for two conditions and an F-statistic for multiple conditions. Finally, VAN estimates the *p*-value for the test statistic using a permutation test and the user can specify the number of permutations to be performed (refer Additional file 1, VAN User Guide Section 11: Measures of association, for a detailed description of the test statistics and permutation tests). A small *p*-value provides evidence that a network module is dysregulated.

### Meta-analysis of network modules

VAN also enables the identification of the subset of modules for which dysregulation (in terms of gene expression correlation with interaction partners) is reproducible across independent cohorts and/or interaction networks (Additional file 1, VAN User Guide Section 5. Meta-analysis of multiple datasets – an example). There are many publicly-available PPI and microRNA-gene interaction networks of varying quality and coverage (discussed in more detail in [13]). As such, VAN explicitly refrains from imposing a specific network on users. Given that there is an extensive and well-documented lack of overlap in the interaction information among network databases [14-18], even for a single gene expression dataset, the set of dysregulated modules is likely to vary from one network dataset to another. This necessitates a meta-analysis of the results obtained using multiple datasets to identify the candidate modules for downstream analysis and/or validation. VAN provides the function *summarizeHubData* for meta-analysis and currently this function supports two methods – Fisher’s combined test and RankProd [19]. In Fisher’s combined test, the overall *p*-value for a network module being dysregulated is computed using the test statistic $-2\sum_{i=1}^{N}\ln p_{i}$. Here, *N* denotes the number of transcriptomics dataset and interaction network combinations and *p*_i_ denotes the probability (of the module being dysregulated) obtained using the i^th^ combination. In contrast, RankProd computes the overall *p*-value using the rank of the network module in each of the *N* combinations; a network module that is consistently ranked high will have a low overall *p*-value.

### ID mapping

An integrative analysis of transcriptomics and PPI data requires the two types of data to map to a common identifier (Additional file 1, Section 6: Generating microRNA-target or protein-protein interaction interactome and Section 8: Conversion of gene symbols to Entrez IDs). In practice, many PPI networks are based on UniProt identifiers whereas transcriptomics data are based on Entrez identifiers or NCBI gene symbols. For ease-of-use, the VAN package automatically maps the various identifiers to one another. VAN provides two functions – *generatePpiMap* and *generateMicroRnaMap* – for creating the input interaction network data. The former function transforms PPI data available in MITAB-lite format (*e.g.*, data downloaded from the protein interaction source iRefWeb [20]), which contains the UniProt identifiers of interacting proteins, into hub-interactor pairs such that the pairs correspond to Entrez identifiers or gene symbols. The latter function generates microRNA-target gene pairs using the TargetScan [21,22] or Microcosm [23,24] databases.

### Network visualization

**Figure 2:**
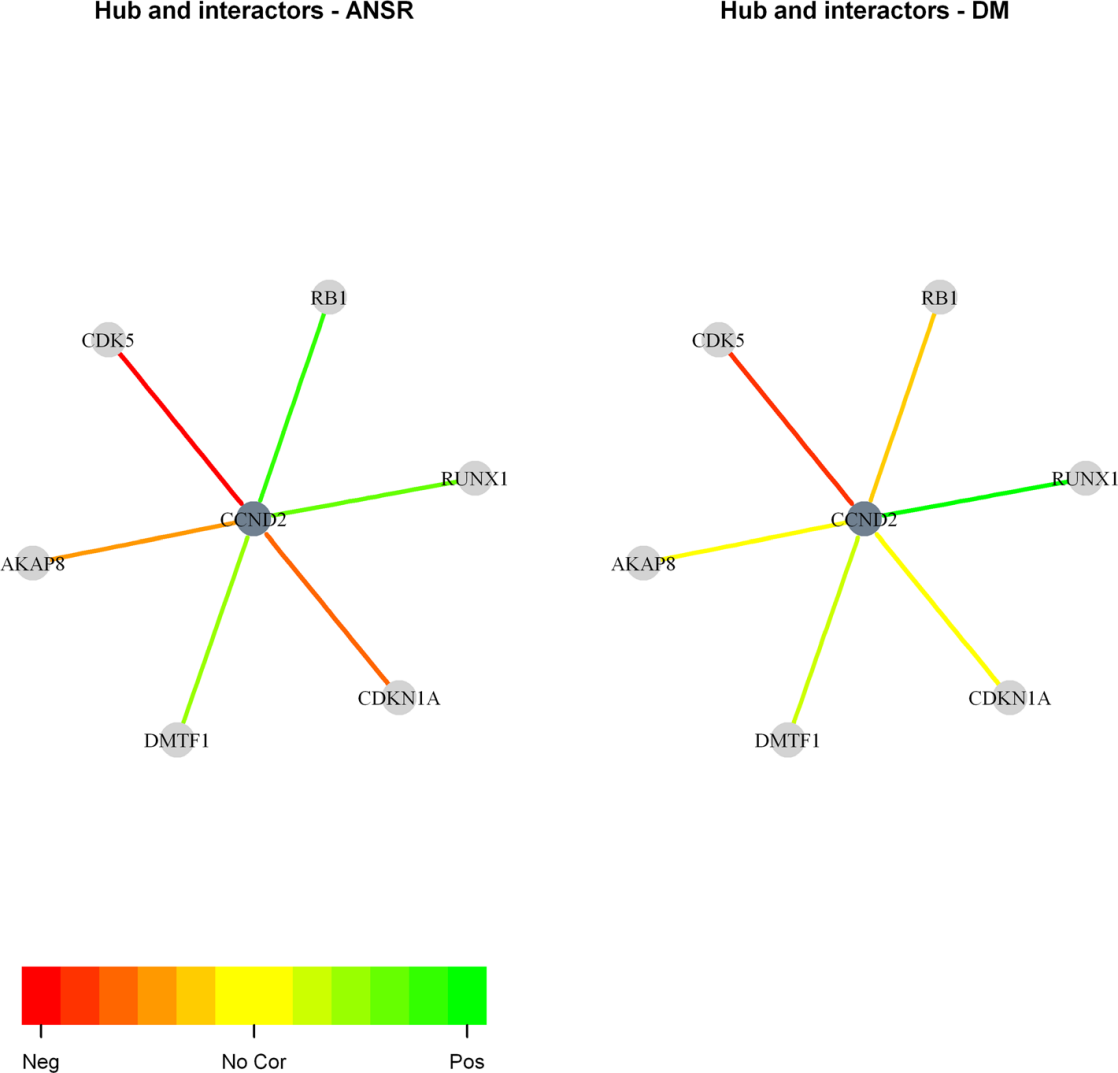
**Network module visualization graph generated in R for the melanoma dataset.** Network interpretation is greatly aided by visualization. Therefore, VAN provides options to visualise individual hubs of interest (in dark grey) linked to their interaction partners (in light grey) via color-coded, undirected edges that are weighted with the co-expression correlation value for a given state as shown here (see also Additional file 1, VAN User Guide Section 4: Option 1: Visualization in R). In this example, we analysed transcriptomic data in metastatic melanoma [28] in the context of a protein-protein interaction network downloaded from the Human Protein Reference Database [29] (refer to Implementation for details). The hub, CCND2, was one of 81 hubs showing significant (*p*-value < 0.05) differences in the average gene expression correlation with respect to its interaction partners. The graph on the left hand side of the figure displays the gene expression correlation coefficients determined for patients alive with no sign of relapse (ANSR) more than 4 years after resection of metastatic disease while the graph on the right hand side shows the same for patients who died from melanoma (DM) within 12 months. The colour scale ranges from red (strong negative correlation of expression) through yellow (no correlation) to green (strong positive correlation).

Network interpretation is greatly aided by visualization [25]. Therefore, VAN provides the function *visualizeNetwork* for plotting the strength of correlation between a hub and each of its interactors via color-coded undirected edges (Figure 2). This tool is available for an analysis involving two conditions (Additional file 1, VAN User Guide Section 4: Option 1: Visualization in R).

For a global visualization of networks of interest, VAN also provides an output file that is directly importable into Cytoscape [26] a popular network visualization tool. To aid with network comprehension in Cytoscape, we also provide an example layout and a 'color-blind safe’ edge palette (created with the aid of ColorBrewer 2.0 and Color Universal Design). The edge palette is supplied as a 'Vizmap property file’ (ExampleVisualStyle.props) and can be directly imported into Cytoscape, as described in the VAN User Guide (Additional file 1). This tool is applicable to the visualization of two or more conditions of interest (Additional file 1, VAN User Guide Section 4: Option 2: Visualization in Cytoscape).

### Extended analysis of cancer datasets

The function *obtainCancerInfo* is used to map the hubs from significantly perturbed network modules to an externally curated catalogue of genes, the cancer Gene Census [27], that are already causally associated with cancer(s). Section 6 of the VAN user guide (Combining output data with known cancer annotation) provides additional details of this aspect of the software which is also illustrated in the example below.

## Implementation

### An example PPI network analysis

**Figure 3:**
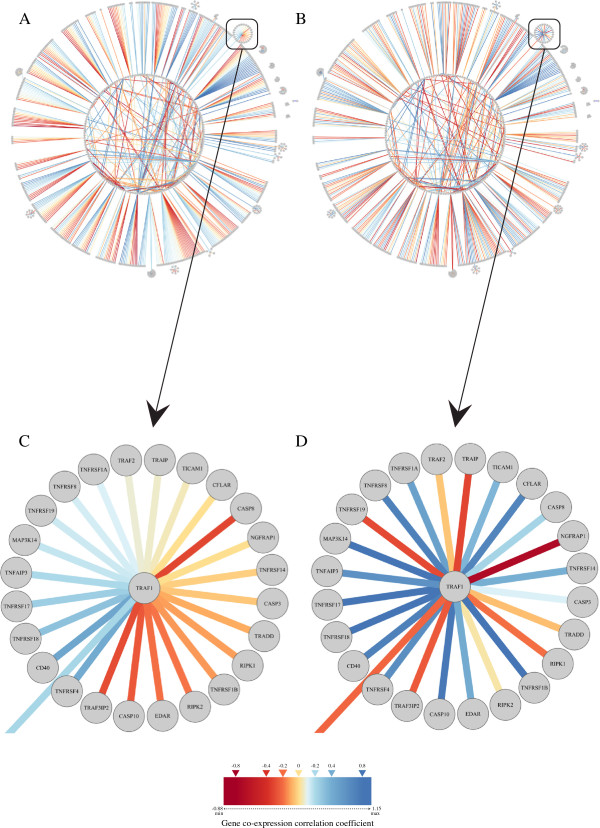
**Network module visualization graphs generated in Cytoscape for the melanoma dataset.** For a global impression of network modules of interest, VAN generates output files that are directly importable into Cytoscape [26], together with a 'color-blind safe’ edge palette file (ExampleVisualStyle.props) and suggested layout protocol (see also Additional file 1, VAN User Guide Section 4: Option 2: Visualization in Cytoscape). As described in Figure 2, we used VAN to analyse gene expression data in metastatic melanoma from Mann *et al.*[28] in the context of a protein-protein interaction network downloaded from the Human Protein Reference Database [29]. The coordination of gene expression among patients with a survival time greater than four years (Figure 3**A**) and patients not surviving beyond 12 months (Figure 3**B**) is affected as indicated by the changes in edge colour. Figures 3**C** and 3**D** zoom in on the same subsets of 3**A** and 3**B**, respectively and show in more detail the significant (*p*-value < 0.05) disruption in the coordination of gene co-expression for the hub *TRAF1* and its interaction partners. Our example visualisation protocol can be applied to two or more conditions (not shown here) and the Cytoscape platform provides dynamic zooming to allow focus on one, or a few, hubs of interest (**C** and **D**).

We used VAN to identify potentially dysregulated modules in relation to patient clinical outcome in disseminated melanoma. For this purpose, we analyzed a publicly available metastatic melanoma gene expression dataset [28] in the context of a PPI network from the Human Protein Reference Database (HPRD, Release 9, April 13, 2010) [29]. The gene expression data corresponded to 45 patients that were split into two groups based on survival time (greater than four years and less than one year). The HPRD PPI network was manually filtered to include only direct, physical PPIs where data were strictly taken only from normal human tissues, denoted as *in vivo* (*vv*). The VAN-based analysis comprised multiple steps. Firstly, we identified hubs and their interaction partners that were present in both the expression and network datasets. Secondly, we evaluated the modules (hubs with at least five interaction partners) for potential dysregulation. Of the 1649 modules evaluated, 81 were potentially dysregulated (*p*-value < 0.05). The resulting network was visualized using two separate platforms, R (Figure 2) as well as Cytoscape (7) (Figure 3), both of which are facilitated by VAN (see Methods) Finally, we searched the cancer Gene Census [27] database to determine whether one or more of the 81 hubs (associated with the dysregulated modules) have previously been causally implicated in cancer(s). We observed that 10 of the 81 hubs – CCND2, CCND3, FANCA, FANCD2, GATA2, KIF5B, LMO2, RET, VHL, WAS (Table 1) have at least one such relationship. Thus, the combination of prior knowledge about mutations with global gene expression data and PPI networks can generate a biologically meaningful context, in this case by pointing to where further mutation discovery could be focused.

**Table 1:** ^**^**^**Cancer Gene Census **[27]** information for the dysregulated hubs identified in the melanoma cancer dataset**

**Symbol**	**CCND2**	**CCND3**	**FANCA**	**FANCD2**	**GATA2**	**KIF5B**	**LMO2**	**RET**	**VHL**	**WAS**
**P-value**	0.04	0.033	0.021	0.036	0.015	0.047	0.016	0.001	0.019	0.01
**Name**	cyclin D2	cyclin D3	Fanconi anemia, complementation group A	Fanconi anemia, complementation group D2	GATA binding protein 2	kinesin family member 5B	LIM domain only 2 (rhombotin-like 1) (RBTN2)	ret proto-oncogene	von Hippel-Lindau syndrome gene	Wiskott-Aldrich syndrome
**Gene ID**	894	896	2175	2177	2624	3799	4005	5979	7428	7454
**Chr**	12	6	16	3	3	10	11	10	3	X
**Chr Band**	12p13	6p21	16q24.3	3p26	3q21.3	10p11.22	11p13	10q11.2	3p25	Xp11.23-p11.22
**Cancer Somatic Mut**	yes	yes	NA	NA	yes	yes	yes	yes	yes	NA
**Cancer Germline Mut**	NA	NA	yes	yes	NA	NA	NA	yes	yes	NA
**Tumour Types Somatic Mutations**	NHL,CLL	MM	NA	NA	AML(CML blast transformation)	NSCLC	T-ALL	medullary thyroid, papillary thyroid, pheochromocytoma, NSCLC	renal, hemangioma, pheochromocytoma	NA
**Tumour Types Germline Mutations**	NA	NA	AML, leukemia	AML, leukemia	NA	NA	NA	medullary thyroid, papillary thyroid, pheochromocytoma	renal, hemangioma, pheochromocytoma	lymphoma
**Cancer Syndrome**	NA	NA	Fanconi anaemia A	Fanconi anaemia D2	NA	NA	NA	Multiple endocrine neoplasia 2A/2B	von Hippel-Lindau syndrome	Wiskott-Aldrich syndrome
**Tissue Type**	L	L	L	L	L	E	L	E, O	E, M, O	L
**Cancer Molecular Genetics**	Dom	Dom	Rec	Rec	Dom	Dom	Dom	Dom	Rec	X-linked recessive
**Mutation Type**	T	T	D, Mis, N, F, S	D, Mis, N, F	Mis	T	T	T, Mis, N, F	D, Mis, N, F, S	Mis, N, F, S
**Translocation Partner**	IGL@	IGH@	NA	NA	NA	RET, ALK	TRD@	H4, PRKAR1A, NCOA4, PCM1, GOLGA5, TRIM33, KTN1, TRIM27, HOOK3, KIF5B, CCDC6	NA	NA
**Other Germline Mut**	NA	NA	NA	NA	NA	NA	NA	yes	NA	NA
**Other Syndrome Disease**	NA	NA	NA	NA	NA	NA	NA	Hirschsprung disease	NA	NA

^Abbreviations: *AML*; acute myelogenous leukemia, *CLL*; chronic lymphatic leukemia, *CML*; chronic myeloid leukemia, *D*; large deletion, *Dom*; dominant, *E*; epithelial, *F*; frameshift, *L*; leukaemia/lymphoma, *M*; mesenchymal, *Mis*; Missense, *MM*; multiple myeloma, *N*; nonsense, *NHL*; non-Hodgkin lymphoma, *NSCLC*; non small cell lung cancer, *O*; other, *Rec*; reccesive, *S*; splice site, *T*; translocation, *T-ALL*; T-cell acute lymphoblastic leukemia, *Chr*; Chromosome, *Mut*; Mutation, *NA*; No data or not applicable.

### Additional examples

In addition to the analysis performed herein, further examples of each of VAN’s functions are provided in the Additional file 1 as part of the VAN User Guide. Section 2 of the user guide describes a number of example gene expression, interactome and VAN output datasets (also refer to Section 7 of the user guide for input data formats). Section 3 of the user guide contains the R code for: 1) analyzing gene expression data (comprising two conditions) with a PPI dataset; 2) analyzing gene expression and microRNA expression data (comprising two conditions) with a microRNA-target interactome; and, 3) analyzing gene expression data (comprising more than two conditions) with a PPI dataset or microRNA-target interactome. Section 5 of the user guide contains example code for meta-analysis.

## Conclusions

Integrative -omics approaches are increasingly popular and will become standard practice in the analysis of complex diseases [1]. Our open source R software package, VAN, provides one possible application of this paradigm. By integrating -omics data with network and mutation data, VAN has the potential to identify network modules (and hubs) of biological relevance to complex human diseases. Although the resulting models of network module dysregulation are largely explanatory/descriptive rather than mechanistic, they do have the potential to highlight dysfunctional pathways, network-centric candidate biomarkers, and/or therapeutic target networks [1,30]. Given that VAN enables the testing of modules for dysregulation based on two or more conditions, it is also suitable for the examination of changes across developmental timelines.

## Availability and requirements

Package name: VAN.

Package repository: sourceforge.

URL for downloading the package: https://sourceforge.net/p/variabilityanalysisinnetworks/wiki/Home/

Operating system(s): Platform independent.

Programming language: R.

Other requirements: R version 2.15.1 or higher.

License: GNU Lesser GPL.

Any restrictions to use by non-academics: None.

The sourceforge project repository for VAN contains –

i. User guide: A step-by-step guide for installing and executing the R package. The user guide also contains example code for data analysis and visualization.

ii. Package functions: A pdf file containing an exhaustive list of all the functions (along with their input and output parameters) available for data analysis and visualization.

iii. R package: The R packages are available for execution on Microsoft Windows©, UNIX, and Mac OS X.

iv. Example dataset: A collection of input and output files for executing the data analysis and visualization examples provided in the user guide.

v. Source code: VAN’s source code.

## Abbreviations

PPI: Protein-protein interaction; VAN: Variability analysis in networks.

## Competing interests

The authors declare that they have no competing interests.

## Authors’ contributions

Conception, design, programing and testing of VAN (VJ/YHY); Major contributions to the design and testing of VAN (VJ/SJS); Writing the manuscript (VJ/SJS/MW); Writing the User Guide (VJ/SJS); Figure preparation and development (VJ/SJS/YHY/MW); Administrative tasks (VJ/SJS); Supervision of data analysis (YHY); Overall scientific direction of the project and final approval of the manuscript (YHY/MW/GJM).

## Supplementary Material

Additional file 1 VAN Package User Guide Version 1.0. Click here for file
