# Supplementary material for: VAN: an R package for identifying biologically perturbed networks via differential variability analysis
Source: BMC Res Notes. 2013 Oct 25;6:430. doi: 10.1186/1756-0500-6-430 (PMC4015612; doi:10.1186/1756-0500-6-430)
Supplement: Additional file 1 — VAN Package User Guide Version 1.0. [file 1756-0500-6-430-S1.pdf]

VAN Package

User Guide Version 1.0

Table of Contents

Topic	Page no
1. Installation instructions	2
2. Example dataset	4
3. Example analysis<br>▪ Protein-protein interactome<br>▪ MicroRNA-target interactome	5
4. Network visualization using R or Cytoscape – an example	7
5. Meta-analysis of multiple datasets – an example	9
6. Generating microRNA/protein based interactome – an example	10
7. Understanding input data and parameters	11
8. Conversion of gene symbols to Entrez Ids	15
9. Understanding output data	16
10. Combining output data with known cancer annotation	17
11. Measures of association	18
12. References	20

Section 1: Installation instructions

Packages to download

- For Windows
    - VAN_1.0.0.zip
    - VANData_1.0.0.zip

- For Unix
    - VAN_1.0.0.tar.gz
    - VANData_1.0.0.tar.gz

- For Mac
    - VAN_1.0.0.tgz
    - VANData_1.0.0.tgz

Example dataset to download

- Example_DataSet.zip

Installation Steps

1. Download and install R version 2.15.1 or higher from the website http://www.r-project.org

2. At the R command prompt type

```
chooseCRANmirror()
## Select one of the options from the pop-up menu, e.g. Australia (Canberra)

setRepositories()
## Select the following three options from the pop-up menu –
## CRAN, BioC software, BioC annotation

install.packages("annotate")
install.packages("doParallel")
install.packages("marray")
install.packages("xlsx")
install.packages("igraph")
install.packages("org.Hs.eg.db")
install.packages("qvalue")
```

3. **For Windows users**, assume that the VAN and VANData packages – VAN_1.0.0.zip and VANData_1.0.0.zip, are saved in the directory C:/My_Packages. Now, at the R command prompt type

```
setwd("C:/My_Packages")
## Note that the separator has to be "/" and not "\"
## for example, setwd("C:\My_Packages") will result in an error message

install.packages("VANData_1.0.0.zip", repos=NULL)
install.packages("VAN_1.0.0.zip", repos=NULL)
```

For Mac users, set the working directory using the appropriate file path syntax. Next, install the following packages

```
install.packages("VANData_1.0.0. tgz", repos=NULL)
install.packages("VAN_1.0.0. tgz", repos=NULL)
```

For Unix users, set the working directory using the appropriate file path syntax. Next, install the following packages

```
install.packages("VANData_1.0.0. tar.gz", repos=NULL)
install.packages("VAN_1.0.0. tar.gz", repos=NULL)
```

Section 2: Example dataset

The Example_DataSet.zip contains –

1. *Gene_Expr_Two_Conditions.txt*: An example gene expression dataset with samples corresponding to two conditions – StateA and StateB

2. *Gene_Expr_Four_Conditions.txt*: An example gene expression dataset with samples corresponding to four conditions – StateA, StateB, StateC, and StateD.

3. *Micro_Expr_Two_Conditions.txt*: An example microRNA expression dataset with samples corresponding to two conditions – StateA and StateB.

4. *PPI_Map.txt*: An example protein-protein interactome file.

5. *Mirnome_Map.txt*: An example microRNA based interactome file.

6. *MiTabLite_Example.txt*: An example MiTabLite file downloaded from the website http://wodaklab.org/iRefWeb/search/index [1]

7. *Gene_Output_1.txt, Gene_Output_2.txt, Gene_Output_3.txt*: Three example output files; each file contains the names of hubs and, for a given hub, the number of interactors and the p-value for the test of significance. The test corresponds to the null hypothesis that there is no change in association between a hub and its interactors across biological states.

8. *Gene_Output_1_Cor.txt*: An example output file containing the correlations for all hub-interactor pairs in two states, namely StateA and StateB.

9. *Gene_Output_2_Cor.txt*: An example output file containing the correlations for all hub-interactor pairs in four states, namely StateA, StateB, StateC, and StateD.

10. *Micro_Output_1_Cor.txt*: An example output file containing the correlations for all microRNA-target gene pairs in two states, namely StateA and StateB.

11. *Cancer_Gene_Census.xls*: An example file downloaded from the website http://www.sanger.ac.uk/genetics/CGP/Census. This file contains the catalogue of genes that have been causally linked to cancer(s).

12. *ExampleVisualStyle.props*: An example *Vizmap Property File* for use with Cytoscape [2] to visualize the changes in hub-interactor associations across biological states. This property file is independent of the dataset being analyzed, which implies that the same property file can be used for visualizing any dataset.

Section 3: Example analysis

For all the analyses, we assume that example dataset (Section 2) is saved in the folder *C:/My_Packages*. We also assume that the VAN package has been loaded and the current working directory has been set appropriately as shown below –

Load the VAN package and set the working directory

At the R command prompt type

```
setwd("C:/My_Packages")
## The above command is for Windows users
## Unix and Mac users should set the working directory using the appropriate file path syntax
library(VAN)
```

The *library* command loads VAN and some additional packages (refer Section 1: *Installation Steps*) into memory. If these packages were developed using a version of R that is different from the version installed on the user's computer, warning messages may be generated. However, these warning messages will not affect the execution of the program.

1. **Combining gene expression data with a protein-protein interaction (PPI) dataset – Two conditions**

```
identifySignificantHubs(exprFile = "Gene_Expr_Two_Conditions.txt"
            , labelIndex = 1
            , mapFile = "PPI_Map.txt"
            , outFile = "Test_Output_PPI.txt"
            , randomizeCount = 10)
```

For an explanation of input/output data, refer sections 7 and 9. If only a subset of the hubs present in the input *PPI_Map.txt* file are of interest, then use the input parameter *hubVect* as shown below

```
identifySignificantHubs(exprFile = "Gene_Expr_Two_Conditions.txt"
                 , labelIndex = 1
                 , mapFile = "PPI_Map.txt"
                 , outFile = "SubTest_Output_PPI.txt"
                 , randomizeCount = 10
                 , hubVect = c("ABL1", "GRB2"))
```

Now, the output file *SubTest_Output_PPI.txt* only contains the results for the two user-defined hubs, namely, ABL1 and GRB2.

2. **Combining gene expression and microRNA expression data with a microRNA-target interactome – Two conditions**

```
identifySignificantHubs(exprFile = c("Gene_Expr_Two_Conditions.txt",
```

```
                                    "Micro_Expr_Two_Conditions.txt")
                , labelIndex = 1
                , mapFile = "Mirnome_Map.txt"
                , outFile = "Test_Output_Mirnome.txt"
                , randomizeCount = 10)
```

The order of the two expression data files is important. As shown in the above example, the first file should correspond to gene expression data and the second file to microRNA expression data.

3. **Combining gene expression with a protein-protein interaction dataset – Multiple conditions**

```
identifySignificantHubs(exprFile = "Gene_Expr_Four_Conditions.txt"
                , labelIndex = 1
                , mapFile = "PPI_Map.txt"
                , outFile = "Test_Output_PPI_Four_Cond.txt"
                , randomizeCount = 10
                , assocType = "FSTAT")
```

4. **Combining gene expression and microRNA expression data with a microRNA-target interactome – Multiple conditions**

   If multiple conditions are to be evaluated for a combination of gene and microRNA expression data, then the *exprFile* input parameter should contain two file names, as shown in Example 2.

Note: In the above examples we set randomizeCount=10 for quick execution. We note that this parameter should be set to 1000 during an actual analysis. For an explanation of the output files, refer Section 9.

Section 4: Network Visualization using R and Cytoscape – An example

We provide two options for visualizing the changes in associations between a protein/microRNA hub and its interactors. Typically, the input file for data visualization will correspond to the output correlation file generated by the function *identifySignificantHubs* (Section 3). For example, if the user was interested in visualizing the hub-interactor correlations corresponding to the analysis performed in Example 1 of Section 3, then the input file will be *Test_Output_PPI_Cor.txt*. However, to illustrate our data visualization function, we consider input files that are already available in the Example dataset (Section 2).

Option 1: Visualization in R

For an analysis involving two conditions, we can visualise a single hub of interest together with its interactors, *e.g.* the gene "ABL1" along with its interactors, by typing at the R command prompt

```
visualizeNetwork(inputFile="Gene_Output_1_Cor.txt"
        , inputHub="ABL1"
        , paletteVector=c("red", "yellow", "green"))
```

For an explanation of the data file to be provided as input, refer Section 9. Similarly, for an example visualization of a microRNA and its interactome, at the R command prompt type

```
visualizeNetwork(inputFile="Micro_Output_1_Cor.txt"
        , inputHub="hsa-miR-551a"
        , paletteVector=c("red", "yellow", "green"))
```

For an analysis involving multiple conditions, we can visualise a single hub of interest together with its interactors for exactly two conditions, *e.g.* the gene "ABL1" along with its interactors in States B and C, by typing at the R command prompt

```
visualizeNetwork(inputFile="Gene_Output_2_Cor.txt"
        , inputHub="ABL1"
        , paletteVector=c("red", "yellow", "green")
        , condVector = c("StateB", "StateC"))
```

Option 2: Visualization in Cytoscape

For visualization in Cytoscape, we can use (i) the output file containing the hub-interactor pairs or (ii) a subset of this file. The advantage of using the subset is that it allows us to focus on only the significant hubs. To obtain the subset, at the R command prompt type

```
obtainPairSubset(filePrefix="Gene_Output_1"
            , useAdjustedProb=FALSE
            , probThresh=0.05)
```

The above command generates an output file "*Gene_Output_1_Cor_Signif.txt*". This file contains the hub-interactor pairs for only those hubs which have an unadjusted p-value less than 0.05

We provide an example layout file and a 'color-blind safe' edge palette (created with the aid of *http:www.//colorbrewer2.org/* and *http://jfly.iam.u-tokyo.ac.jp/color/*) for visualization using Cytoscape. For an analysis involving two conditions (*e.g.*, StateA and StateB), the steps to be followed are:

1. Download and install Cytoscape from the website *http://www.cytoscape.org/download.html*

2. From the Cytoscape Desktop menu bar select "File", "Import", "Network from Table (Text/MS Excel)"

3. At the "Import Network and Edge Attributes from Table" window:

    a. Select the input file: this will be the VAN output file *Gene_Output_1_Cor_Signif.txt*
    b. At the "Advanced" options, check "Show Text File Import Options"
    c. At the "Text File Import Options, uncheck the "Delimiter" "Space"
    d. At "Attribute Names" check "Transfer first line as attribute names"
    e. At the "Interaction Definition" options, select "Column 1" as the Source Interaction and Column 2 as the Target Interaction
    f. At the "Preview" click on Columns 3 to 5 to activate import of these data

4. From the menu bar select "Layout", "Cytoscape Layouts", "Circular Layout"

5. From the menu bar select "File", "Import", "Vizmap Property File)" and select the input file: "*ExampleVisualStyle.props*"

6. At the Cytoscape Desktop Control Panel, click on the VizMapper™ tab and select "example visual style" as the Current Visual Style

7. At the Visual Mapping Browser, select "StateA" in the drop down box at the "Edge Color" field. Next, select "StateB" to visualize how the network co-expression organisation varies as a function of the second condition (StateB) compared with the first (StateA).

For multiple conditions, the above procedure is followed with additional Columns activated at the data import step (refer to 3f, above). This should enable multiple States to be available for viewing as described at 7, above.

Section 5: Meta-analysis of multiple datasets – An example

To combine the results obtained using multiple datasets, we have implemented two meta-analysis methods – Fisher's combined test and RankProd [3]. Since both methods assume independence of datasets, the output *p*-values should be interpreted with caution if the same expression dataset was combined with multiple protein or microRNA interactomes.

Typically, the input files will correspond to the output files generated by the function *identifySignificantHubs* for different interaction networks or expression datasets. However, to illustrate the meta-analysis feature of our package, we use the output files provided in the Example dataset (Section 2). At the R command prompt type

```
inputFileVect = c("Gene_Output_1.txt", "Gene_Output_2.txt", "Gene_Output_3.txt")

## Fisher's combined test
summarizeHubData(fileNames=inputFileVect
            , outFile="Summary_Mann_Fisher.txt"
            , metaAnalysis="Fisher")

## RankProd
## This is the RankProd implementation provided in the R package MADAM (Version
## 1.1)
summarizeHubData(fileNames=inputFileVect
            , outFile="Summary_Mann_RP.txt"
            , metaAnalysis="RankProd"
            , rankProdItr=10)
```

In the above example we set rankProdItr=10 for quick execution. We note that this parameter should be set to 1000 during an actual analysis and for an explanation of the output files, refer Section 9.

Section 6: Generating microRNA-target or protein-protein interaction interactome

a. *Protein-protein interactions*: To generate an input protein interactome file, perform the following steps –
   i. Download a MiTab Lite file from the website http://wodaklab.org/iRefWeb/search/index
   ii. Apply the function *generatePpiMap* to the MiTab Lite file.

   To illustrate this, we use the example file "MiTabLite_Example.txt" described in Section 2. At the R command prompt type

```
generatePpiMap("MiTabLite_Example.txt", "Test_PPI")
```

b. *MicroRNA-target interactions*: To generate an input microRNA interactome file corresponding to TargetScan [4], at the R command prompt type

```
generateMicroRnaMap("Targetscan", "TS_Mirnome")
```

   Similarly, to generate the mapping corresponding to MicroCosm [5], at the R command prompt type

```
generateMicroRnaMap("Microcosm", "MC_Mirnome")
```

In each of the three instances, two output files are generated – one with suffix "Entrez" and the other with suffix "Symb", *e.g.* Test_PPI_Entrez.txt and Test_PPI_Symb.txt The former contains the hub-interactor pairs as Entrez IDs and the latter as gene symbols. During the generation of the interactome files, some error files may also be generated (refer Section 8).

The function *generateMicroRnaMap* relies on the mapping information available in the package VANData. This package is updated periodically to correspond to the most recent releases of TargetScan and MicroCosm databases.

As an alternative to the example input interactomes, a user may provide a query network of his own *e.g.*, based on a set of genes of interest. We note that considerations for network selection are discussed in detail in [6], which also provides several selected examples of interactomes and sub-networks generated by other groups using a multitude of methods including hypothesis-driven and meta-mining approaches *e.g.*, [7-15].

Section 7: Understanding input data and parameters

Input file formats

a. *Expression data*: VAN does not provide any functions for preprocessing user expression data, such as data normalization to remove systematic variation, or other potential steps including data transformation and/or filtering. These functions are widely available elsewhere and users should provide, as input to VAN, an appropriately normalized and filtered data.

   The input expression data file should be a tab-separated text file. The first column should correspond to gene/microRNA names and the remaining columns should correspond to expression values (Figure 1). Therefore, if there are N samples, the number of columns in the input file should be N + 1. Each gene or microRNA name should occur exactly once in the tab-separated file and the gene names could be provided as Entrez IDs or gene symbols. The file should also contain labels for grouping the N samples. For example, if the N samples correspond to two physiological states, say A and B, then an appropriate label should be assigned to each of the N samples. The keyword **LABEL_END** is used to separate the labels from the actual expression values and allows the user to provide, in a single expression data file, multiple ways of grouping the samples (Figure 2). For example, let us assume that the N samples can be grouped based on disease status (say StateA and StateB) and mutation (say Wt and Mut) and we are interested in identifying the enriched hubs in both cases. Instead of generating two gene expression data files, one with labels corresponding to disease status and the other with labels corresponding to mutation status, we can provide the two sets of labels in the same expression data file.

Blank cell

sample labels

	StateA	StateB	StateB	StateA	StateA	StateB
LABEL_END						
A26C3	-0.27	-0.39	1.02	-0.90	0.78	0.50
A2BP1	-0.97	0.63	0.69	1.29	0.95	1.35
A2M	0.68	-0.42	0.09	-0.73	-0.94	1.09

Figure 1: An example input expression data file. Each column represents a sample and has an associated label (StateA or StateB in the present case). The row that contains the keyword LABEL_END should not have any other value. This can be achieved in two ways based on the software used to generate the expression data file – (i) if the data file was generated in MS-Excel, then the remaining cells in the row corresponding to LABEL_END should be left empty, (ii) if the data was generated using a text editor, then

the carriage return (i.e. the key labeled "Enter" on the keyboard) should be pressed immediately after LABEL_END.

	StateA	StateB	StateB	StateA	StateA	StateB	← Set 1
	Wt	Mut	Mut	Wt	Wt	Mut	← Set 2
LABEL_END							
A26C3	-0.27	-0.39	1.02	-0.90	0.78	0.50	
A2BP1	-0.97	0.63	0.69	1.29	0.95	1.35	
A2M	0.68	-0.42	0.09	-0.73	-0.94	1.09	

Figure 2: An example input expression data file with two sets of labels

b. *Interactome data*: The interactome data file is tab-separated and has two columns – the first column corresponds to hubs and the second column to interactors. The first row of the data file is assumed to be the header and is ignored while reading the data. For protein interactomes, the names of hubs and their interactors can be provided as Entrez IDs or gene symbols but not as a combination of the two. For microRNA interactomes, the names of interactors can be provided as Entrez IDs or gene symbols.

Input parameters

We assume that a network module comprises two types of genes – hub and interactor. The hub gene is connected to all the interactor genes and every interactor gene is connected to only the hub gene. The main function for identifying the enriched network modules is *identifySignificantHubs.* This function has 14 input parameters and a detailed description of the parameters is provided in the PDF file ***VAN_Package_Functions.pdf***. Of the 14 input parameters, only four have to be explicitly specified by the user – *exprFile, labelIndex, mapFile,* and *outFile*. The default values for the remaining 10 parameters need to be changed only under certain conditions and below we describe those conditions.

Parameter	*Condition where the default value should be changed*
hubSize = 5	By default, only those network modules that have at least 5 interactors in the expression data set are considered for downstream analysis. This value can be changed to consider more dense or sparse modules.
randomizeCount = 1000	By default 1000 permutations are performed to determine the p-value. The user can select a higher number of permutations, however, it should be noted

	that an increase in the number of permutations will increase the execution time. The number of permutations can also be lowered but is not recommended.
adjustMethod = "BH"	By default, the p-values for modules are adjusted using the Benjamimi-Hochberg (or false discovery rate) adjustment method [16], as implemented in R. Alternatively, the bonferroni adjustment may be used.
assocType = "TCC"	If the number of conditions is two, then the association between a hub-interactor pair can be measured in two ways (refer Section 10). The default option is "TCC" [17] and the other available option is "PCC" (short for Pearson's correlation coefficient)<br><br>If the number of conditions is more than two, then this parameter must be set to "FSTAT".
labelVect = NULL	By default, this value is set to NULL and implies that all the N samples in the expression data are used for measuring association.<br><br>Sometimes one may be interested in evaluating only a subset of conditions present in the expression data. For example, the N samples in the expression data may correspond to Stage 1, Stage 2, and Stage 3 of cancer and one may be interested in only evaluating the samples that correspond to stages 1 and 2. Rather than creating a new expression data file with just two stages, we can use the existing expression data file and provide (as input) the vector of only those conditions that need to be evaluated.
exprDataType = "SYMB"	By default, the gene names in the expression data file are assumed to

	correspond to gene symbols. However, if the gene names correspond to Entrez IDs, this parameter should be changed to "ENTREZ". It should be noted that if two expression data files are provided (one for gene expression and another for microRNAs), then both should contain the same type of gene names *i.e.* IDs should not be mixed.
ppiDataType = "SYMB"	By default, the gene names in the interactome data file are assumed to be gene symbols. If that is not the case, this parameter should be set to "ENTREZ".
outputDataType = "SYMB"	By default, the output files save the hub and interactors as gene symbols. However, the user can choose to save the two as Entrez IDs by setting this parameter to "ENTREZ".
Species = "Human"	Currently only human is supported.
inputCores = 4	This denotes the number of microprocessor cores that are available for executing the code in parallel. The number is decreased automatically if fewer than four cores are available. However, if more than four cores are available, then this number can be increased explicitly.

We note that during the conversion of gene symbols to Entrez Ids, some error files may be generated as described in the next section.

Section 8: Conversion of gene symbols to Entrez IDs

Expression data

If the expression data and interactome data contain gene labels in different formats, *i.e.* one corresponds to Entrez IDs and the other to gene symbols, then the gene symbols are mapped to Entrez IDs. For the mapping process, we utilize the Bioconductor [18] annotation files and, in some instances, the gene symbol to Entrez ID mapping is unavailable. The list of gene symbols that could not be mapped to Entrez IDs is saved in the following files –

a. *Error_Expr.txt*: This file contains the gene symbols that were present in the expression data but could not be mapped to Entrez IDs.

b. *Error_PPI_Hubs.txt*: This file contains the hubs for which the gene symbols could not be mapped to Entrez IDs.

c. *Error_PPI_Int.txt*: This file contains the interactors for which the gene symbols could not be mapped to Entrez IDs

Interactome data

The functions *generatePpiMap* and *generateMicroRnaMap* are used to generate the interactome data files. These functions return the hub-interactor pairs in two formats – one corresponding to Entrez IDs and the other to gene symbols. As mentioned earlier, in some instances, the Entrez ID to gene symbol mapping is unavailable and the Entrez IDs that could not be mapped to gene symbols are saved in the following files –

a. *Error_Mirnome_Generation.txt*: This file contains the microRNA-gene pairs for which the Entrez IDs could not be mapped to gene symbols

b. *Error_PPI_Generation.txt*: This file contains the gene-gene pairs (hub-interactor pairs) for which the Entrez IDs (for at least one of the genes in the pair) could not be mapped to gene symbols.

Section 9: Understanding output data

All the output data files are tab-separated and can be viewed using a text editor or MS Excel.

Enriched Modules

The function *identifySignificantHubs* is used to evaluate the network modules (i.e. hubs and their associated interaction partners) for a given combination of expression and interactome data. This function generates two output files – one containing the p-values and the second containing the hub-interactor associations in each of the conditions. While the name of the first file is explicitly provided as an input parameter by the user, the name of the second file is obtained by adding the suffix "_Cor" to the first file.

To visualize the changes in association between a hub protein/microRNA and its interactors using our R function, the second file ("_Cor") is used. We note that only two conditions can be visualized simultaneously using our R function (refer Section 4). Therefore, if the output file corresponds to multiple conditions, then the two conditions to be plotted have to be explicitly specified by the user (refer Section 4, Option 1). To visualise global changes among significantly enriched protein/microRNA hubs and their interactors, using the Cytoscape [2] program, the second file ("_Cor") is filtered (refer Section 4, Option 2). Both the two- and multiple condition files are suitable for upload and visualisation using Cytoscape.

Meta-analysis

The function *summarizeHubData* is used to perform meta-analysis and aggregate the results obtained using multiple datasets. The meta-analysis output file contains all the modules that were tested for enrichment in at least one of the datasets.

Fisher's combined test: If the meta-analysis method is set to "Fisher", then for a given module, the Fisher's combined test p-value is calculated using only those datasets in which the module was present. The combined p-value is obtained using the unadjusted p-values (for the module) in the individual datasets. This combined p-value is saved as the last column of the meta-analysis output file. We note that the combined p-value provided in the output file is not adjusted for multiple comparisons.

RankProd: If the meta-analysis method is set to "RankProd", then the ranks of the modules in the individual datasets are used to obtain aggregate ranks. If a module was not evaluated in a dataset, its rank is set to K, where K denotes the total number of modules. Also, if in a given dataset, multiple modules have the same p-value, then they are assigned the same rank. The unadjusted and FDR-adjusted RankProd p-values are saved as the last two columns in the meta-analysis output file.

Section 10: Combining output data with known cancer annotation

In case of cancer-related datasets, to facilitate the biological interpretability of the enriched hubs, we provide a function *obtainCancerInfo*. This function maps the hubs (corresponding to enriched modules) to the catalogue of genes already causally associated with cancer(s), provided the catalogue file is provided as an input parameter in Excel format. For example, to determine whether hubs (associated with enriched modules) in the output file *Gene_Output_1.txt* (Section 2) correspond to known cancer genes, at the R command prompt type

```
obtainCancerInfo(hubFile = "Gene_Output_1.txt"
            , cancerAnnotationFile = "Cancer_Gene_Census.xls"
            , outFile = "Hub_CIC_Info.txt")
```

The output file *Hub_CIC_Info.txt* contains hubs (with unadjusted p-value < 0.05) that map to known cancer genes.

Unlike the microRNA interactomes which are updated regularly in the *VANData* package, the *Cancer_Gene_Census* file is for example purposes only and is not updated. Therefore, we encourage the user to download the most up-to-date version of the annotation file from the website http://www.sanger.ac.uk/genetics/CGP/Census/ prior to executing this R function.

Section 11: Measures of association

Inside our R package, every row of the expression data is median-centered and its variance is set to one prior to the calculation of the association measure. Before we describe the various association measures implemented in our package, we introduce some notation. We denote a hub (microRNA or gene) as *u* and its set of interactors as R such that $r \in$ R is an individual element (or interactor) in the set R. We denote the number of biological states by *N* and the choice of *N* determines the association measure.

Number of conditions is two

If *N* = 2, then the user can select Taylor's correlation coefficient (TCC) [17] or Pearson's correlation coefficient (PCC) as the association measure.

1. **TCC**: For a given hub-interactor pair (i.e. a *u*-*r* pair)

$$\rho^r_{B1} = \frac{\sum_{i \in B1} (X_{u_i} - \overline{X_u})(X_{r_i} - \overline{X_r})}{(n_{B1} - 1) s_{u_{B1}} s_{r_{B1}}} \quad (1)$$

and

$$\rho^r_{B2} = \frac{\sum_{i \in B2} (X_{u_i} - \overline{X_u})(X_{r_i} - \overline{X_r})}{(n_{B2} - 1) s_{u_{B2}} s_{r_{B2}}} \quad (2)$$

where B1 and B2 denote the two biological states, n_{B1} and n_{B2} denote the number of samples in B1 and B2, respectively, X_{u_i} and X_{r_i} denote the expression value for the hub and the interactor, respectively, in the ith sample, $s_{u_{B1}}$ and $s_{r_{B1}}$ denote the sample standard deviation for the hub and interactor, respectively, in B1, $s_{u_{B2}}$ and $s_{r_{B2}}$ denote the sample standard deviation for the hub and interactor, respectively, in B2, ρ^r_{B1} and ρ^r_{B2} denote the correlation coefficient in B1 and B2, respectively, for the *u*-*r* pair. Also, $\overline{X_u}$ and $\overline{X_r}$ denote the average expression value for *u* and *r*, respectively, over all samples, i.e. over $n_{B1} + n_{B2}$ samples. Now,

$\Delta\rho^r = \rho^r_{B1} - \rho^r_{B2}$ and

$$\overline{\rho} = \frac{\sum_{r \in R} | \Delta\rho^r |}{N_R - 1}$$

where N_R is the number of interactors in the set R.

We test the null hypothesis that the average change in association between a hub and its interactors (i.e. $\overline{\rho}$) is not stronger than that by chance. For this purpose, we randomly assign the samples to B1 and B2 and recalculate $\overline{\rho}$. We denote the recalculated $\overline{\rho}$ as $\overline{\rho}*$ and perform the random assignment of samples N_{perm} times. We estimate the p-value as the proportion of times $\overline{\rho}*$ is at least as large as $\overline{\rho}$. If the p-value is less than a user-defined threshold value, say 0.05, then we consider the change in association between a hub and its interactors to be statistically significant.

2. **PCC**: The PCC differs from TCC in the estimation of ρ^{r}_{B1} and ρ^{r}_{B2}. Unlike Equations (1) and (2), for PCC

$$\rho^{r}_{B1} = \frac{\sum_{i \in B1} (X_{u_i} - \overline{X_{u_{B1}}})(X_{r_i} - \overline{X_{r_{B1}}})}{(n_{B1} - 1) s_{u_{B1}} s_{r_{B1}}}$$

and

$$\rho^{r}_{B2} = \frac{\sum_{i \in B2} (X_{u_i} - \overline{X_{u_{B2}}})(X_{r_i} - \overline{X_{r_{B2}}})}{(n_{B2} - 1) s_{u_{B2}} s_{r_{B2}}}$$

where $\overline{X_{u_{B1}}}$ and $\overline{X_{u_{B2}}}$ denote the average expression value for u in B1 and B2, respectively. Similarly, $\overline{X_{r_{B1}}}$ and $\overline{X_{r_{B2}}}$ denote the average expression value for r in B1 and B2, respectively.

Number of conditions is greater than two

For every biological state, we calculate the PCC between a hub u and its interactors $r \in$ R. Thus we obtain a matrix M with N_R rows (corresponding to the number of interactors) and N columns (corresponding to the number of biological states). We use the matrix M to calculate the F-statistic [19]. We denote the observed F-statistic as f^{obs} and test the null hypothesis that f^{obs} value is not larger than that obtained by chance. For this purpose we perform a permutation test similar to that described earlier for TCC.

Section 12: References

1. Turner B, Razick S, Turinsky AL, Vlasblom J, Crowdy EK, Cho E, Morrison K, Donaldson IM, Wodak SJ: **iRefWeb: interactive analysis of consolidated protein interaction data and their supporting evidence**. *Database-the Journal of Biological Databases and Curation* 2010.
2. Smoot ME, Ono K, Ruscheinski J, Wang PL, Ideker T: **Cytoscape 2.8: new features for data integration and network visualization**. *Bioinformatics* 2011, **27**(3):431-432.
3. Hong FX, Breitling R, McEntee CW, Wittner BS, Nemhauser JL, Chory J: **RankProd: a bioconductor package for detecting differentially expressed genes in meta-analysis**. *Bioinformatics* 2006, **22**(22):2825-2827.
4. Garcia DM, Baek D, Shin C, Bell GW, Grimson A, Bartel DP: **Weak seed-pairing stability and high target-site abundance decrease the proficiency of lsy-6 and other microRNAs**. *Nature Structural & Molecular Biology* 2011, **18**(10):1139-U1175.
5. Griffiths-Jones S, Saini HK, van Dongen S, Enright AJ: **miRBase: tools for microRNA genomics**. *Nucleic Acids Research* 2008, **36**:D154-D158.
6. Schramm S-J, Jayaswal V, Goel A, Li SS, Yang YH, Mann GJ, Wilkins MRUrMina-oragttpol-snfah-tdP: **All networks are wrong, but are any networks useful? Progress in preparing molecular interaction networks for the analysis of human disease.** *Proteomics* 2013, **Under revision**.
7. Yao C, Li H, Zhou C, Zhang L, Zou J, Guo Z: **Multi-level reproducibility of signature hubs in human interactome for breast cancer metastasis**. *BMC Systems Biology* 2010, **4**(1):151.
8. Rual J-F, Venkatesan K, Hao T, Hirozane-Kishikawa T, Dricot A, Li N, Berriz GF, Gibbons FD, Dreze M, Ayivi-Guedehoussou N *et al*: **Towards a proteome-scale map of the human protein-protein interaction network**. *Nature* 2005, **437**(7062):1173-1178.
9. Martha V-S, Liu Z, Guo L, Su Z, Ye Y, Fang H, Ding D, Tong W, Xu X: **Constructing a robust protein-protein interaction network by integrating multiple public databases**. *BMC Bioinformatics* 2011, **12**(Suppl 10):S7.
10. Davis MJ, Shin CJ, Jing N, Ragan MA: **Rewiring the dynamic interactome**. *Molecular Biosystems* 2012, **8**(8):2054-2066.
11. Wang X, Wei X, Thijssen B, Das J, Lipkin SM, Yu H: **Three-dimensional reconstruction of protein networks provides insight into human genetic disease**. *Nat Biotech* 2012, **30**(2):159-164.
12. Sowa ME, Bennett EJ, Gygi SP, Harper JW: **Defining the Human Deubiquitinating Enzyme Interaction Landscape**. *Cell* 2009, **138**(2):389-403.
13. Christianson JC, Olzmann JA, Shaler TA, Sowa ME, Bennett EJ, Richter CM, Tyler RE, Greenblatt EJ, Wade Harper J, Kopito RR: **Defining human ERAD networks through an integrative mapping strategy**. *Nat Cell Biol* 2012, **14**(1):93-105.
14. Bennett EJ, Rush J, Gygi SP, Harper JW: **Dynamics of Cullin-RING Ubiquitin Ligase Network Revealed by Systematic Quantitative Proteomics**. *Cell* 2010, **143**(6):951-965.
15. Behrends C, Sowa ME, Gygi SP, Harper JW: **Network organization of the human autophagy system**. *Nature* 2010, **466**(7302):68-76.
16. Benjamini Y, Hochberg Y: **Controlling the False Discovery Rate - a Practical and Powerful Approach to Multiple Testing**. *Journal of the Royal Statistical Society Series B-Methodological* 1995, **57**(1):289-300.

17. Taylor IW, Linding R, Warde-Farley D, Liu YM, Pesquita C, Faria D, Bull S, Pawson T, Morris Q, Wrana JL: **Dynamic modularity in protein interaction networks predicts breast cancer outcome**. *Nature Biotechnology* 2009, **27**(2):199-204.
18. Gentleman RC, Carey VJ, Bates DM, Bolstad B, Dettling M, Dudoit S, Ellis B, Gautier L, Ge YC, Gentry J *et al*: **Bioconductor: open software development for computational biology and bioinformatics**. *Genome Biology* 2004, **5**(10).
19. Rice JA: **Mathematical Statistics and Data Analysis**, 2 edn. Belmont: Wadsworth Publishing Company; 1995.
